# HROP68: A rare case of medullary pancreatic cancer—characterization and chemosensitivity of the first patient-derived cell line

**DOI:** 10.3389/fonc.2022.1082927

**Published:** 2023-01-20

**Authors:** Jens von den Driesch, Jana Flöttmann, Friedrich Prall, Christina S. Mullins, Michael Linnebacher, Florian Bürtin

**Affiliations:** ^1^ Clinic of General, Visceral, Vascular and Transplantation Surgery, University Medical Center Rostock, University of Rostock, Rostock, Germany; ^2^ Institute of Pathology, University Medical Center Rostock, University of Rostock, Rostock, Germany

**Keywords:** PDAC - pancreatic ductal adenocarcinoma, rare malignancy, medullary adenocarcinoma, precision medicine, patient-derived cell line

## Abstract

**Introduction:**

Medullary pancreatic carcinoma (MPC) is a rare subtype of pancreatic ductal adenocarcinoma. MPCs represent less than 1% of all pancreatic cancers, and, with only 26 cases in the literature, knowledge regarding drug response and treatment outcome is very limited.

**Material and methods:**

We present the case of a 64-year-old male patient with MPC who was treated by left pancreatic resection and adjuvant chemotherapy. Due to local recurrence, the patient underwent intended curative reoperation. From both surgical specimens, patient-derived xenografts (PDXs) and, from the recurrence, a patient-derived cell line (PDCL) were established. We subsequently performed an in-depth characterization of this cell line including phenotypic characterization, surface protein expression, growth, and migratory performance as well as mutational analysis using whole-exome sequencing (WES). Additionally, *in vitro* drug sensitivity toward the standard-of-care chemotherapeutic regimen and selected targeted therapies was evaluated.

**Results:**

The pathological and molecular properties of this rare MPC case observed in the patient’s tumors are preserved in the corresponding PDX and the PDCL of HROP68Tu2. Despite displaying an “immunogenic phenotype” with marked T-cell infiltration and a high-level expression of HLA II and Programmed death-ligand 1 (PD-L1), molecular analysis revealed microsatellite stability but a multitude of mutations affecting KRAS, TP53, KAT6B, FOXG1, RUNX1, and GRIK2 among others. Furthermore, HROP68Tu2 cells were susceptible toward 5-FU, irinotecan, oxaliplatin, gemcitabine, paclitaxel, and erlotinib as single agents, but only a moderate synergistic response was seen to the drugs of the FOLFIRINOX regimen. Even worse, the drugs of the two combinations gemcitabine plus paclitaxel and gemcitabine plus erlotinib showed antagonistic effects. Moreover, lapatinib, PRIMA-Met1, and olaparib selected as targeted therapeutics according to the mutational profiles and protein expression inhibited HROP68Tu2 cells’ growth.

**Conclusion:**

This study illustrates the establishment of the first preclinical MPC models as well as the first in-depth characterization of an MPC PDCL. Since the scientific and clinical knowledge of this rare pancreatic cancer type is very limited, the presented models contribute to a better understanding of MPC and might be a valuable tool for the development of future treatment options.

## 1 Introduction

Pancreatic ductal adenocarcinoma (PDAC) is the fourth leading cause of cancer-related death in the United States and the seventh worldwide (1, 2). Despite an increasing incidence, especially in countries with a high human development index, the prognosis remains dismal with reported 5-year survival rates ranging from 4.2% to 10% (1–3). Since most patients present with advanced cancer stages, only 10%–16% of the patients are eligible for resection, currently the only curative treatment option. Yet, those treated by surgery also show a poor prognosis with a median survival of 18 months and a 5-year survival rate of 15%–25% due to early local recurrence or metastasis (1, 4, 5).

Recent survival improvements are mainly attributed to intensified adjuvant and palliative chemotherapy regimens. In the adjuvant setting, modified FOLFIRINOX, a drug regimen comprised of folinic acid, fluorouracil (5-FU), irinotecan, and oxaliplatin, as well as the combination of gemcitabine with capecitabine has shown improved survival compared to gemcitabine monotherapy (6, 7). In locally advanced or metastatic stages, FOLFIRINOX and gemcitabine plus nanoparticle-bound paclitaxel are first-line treatment options (8). Nevertheless, most PDACs are characterized by a markedly low susceptibility to chemotherapy mediated by a specific tumor microenvironment with a dense desmoplastic reaction and the infiltrates of immune-suppressive cell populations as well as by a low tumor mutational burden (TMB) (9–11). In contrast, pancreatic medullary carcinoma (MPC), a rare subtype of PDAC, appears pathologically cell rich and low in stroma and is often characterized by a high TMB and microsatellite instability (12, 13). To the best of our knowledge, our group established the first patient-derived xenograft (PDX) and patient-derived cell line (PDCL) from a recurrent medullary pancreatic carcinoma, named HROP68Tu2, with distinct clinical, pathological, and molecular characteristics. In addition to the morphological and molecular characterization of the PDCL, the response to therapeutic agents was evaluated *in vitro*. 5-FU, irinotecan, oxaliplatin, gemcitabine, paclitaxel, and erlotinib were tested as single agents and in the clinically established combinations FOLFIRINOX, gemcitabine combined with paclitaxel (GemPac), and gemcitabine plus erlotinib (GemErlo). Since whole exome sequencing (WES) revealed a distinct mutational profile, and the tumor cells expressed high levels of HER2 and EGFR, the targeted drugs lapatinib, olaparib, and PRIMA-Met1 were additionally tested.

## 2 Materials and methods

### 2.1 Patient-derived xenograft and cell line establishment

The collection and processing of tumor tissue have been approved by the institutional review board of the University Medical Center Rostock (A 2018-0054). All animal experiments have been approved by the Landesamt für Landwirtschaft, Lebensmittelsicherheit und Fischerei Mecklenburg-Vorpommern under the registration numbers LALLF M-V/TSD/7221.3-1-007/19.

The tumor processing and establishment of PDX and PDCL were conducted as described previously (14, 15). In brief, after surgical en bloc resection of the tumor, a small tumor piece irrelevant for the evaluation of the resection margin was removed, cleaned, cut into cubes of 3 × 3 × 3 mm under sterile conditions, and cryopreserved immediately. Afterward, tumor specimens were thawed, incubated in Matrigel^®^ (Corning, New York, USA), and subcutaneously implanted into the flanks of NSG mice. After reaching the required tumor size, mice were euthanized and PDX were explanted and cut accordingly for re-engraftment, histological examination, and snap freezing.

For cell line establishment, tumor tissue was mechanically dissected and passed through a 100 µm cell strainer. After centrifugation and resuspension in PBS, cells were seeded in a collagen-coated 6-well plate in a culture medium including antibiotics and antimycotics and incubated at 37°C in a humidified atmosphere of 5% CO_2_. As stable growth was observed, cells were transferred into a 25 cm^2^ culture flask with a standard culture medium (DMEM/F12 (1:1), 5% fetal calf serum (FCS), and 2 mM L-glutamine; all cell culture reagents were from PAN-Biotech, Aidenbach, Germany. Cells were regularly tested for mycoplasma contamination using the PlasmoTest™—Mycoplasma Detection Kit (*In vivo*Gen, San Diego, CA, USA) according to the manufacturer’s protocol.

### 2.2 Short tandem repeat analysis

The concordance of HROP68Tu2 PDCL cells and healthy donor tissue was confirmed by short tandem repeat (STR) analysis as previously described (14). In short, DNA from donor tissue and tumor cells was isolated and the fragments of D5S818, D7S820, D16S539, D13S317, vWA, TPOX, THO1, CSF1PO, and Amelogenin were PCR-amplified with fluorescence-labeled primers. Subsequently, samples were size-separated and analyzed by automated capillary electrophoresis (Thermo Fisher Scientific, Waltham, MA, USA) (14).

### 2.3 Growth and migration

For doubling time determination, 4 × 10^4^ HROP68Tu2 cells were seeded in a coated 24-well plate and allowed to attach for 72 h. To determine the mass of the vital cells, a crystal violet assay was conducted as described previously (16). Cells were stained with 0.2% crystal violet every 24 h in quadruplicates for six consecutive days and absorption was measured at 540 nm using the plate reader Tecan Infinite (Tecan, Männedorf, Switzerland). Migration speed was determined by the scratch assay. Cells grown to 100% confluency in a 6-well plate were switched to a serum-free medium and scratched with a 10 µl pipette tip. Three distances between edges were measured every 24 h for four consecutive days at 10× magnification using an inverted microscope (Carl Zeiss AG, Jena, Germany). The experiment was performed in independent triplicates.

### 2.4 Flow cytometry

The cell surface proteins of HROP68Tu2 cells were analyzed by flow cytometry using FACS Calibur (BD Bioscience, Franklin Lakes, NJ, USA) and open-source FCSalyser software. A panel of FITC-, PE-, and APC-conjugated antibodies was used for direct staining, targeting CD13, CD15, CD29, CD40, CD54, CD58, CD66adecb, CD71, CD86, CD95, HLA class I, HLA class II (Immunotools, Friesoythe, Germany); CD26, CD80, CD178 (eBioscience, Thermo Fisher Scientific, MA, USA); CD152, CD227 (Biolegend, San Diego, CA, USA); CD90 (Dianova, Eching, Germany); and CD326 (Miltenyi Biotech, Bergisch-Gladbach, Germany). Pembrolizumab (anti-PD-1), atezolizumab (anti-PD-L1), cetuximab (anti-EGFR), trastuzumab (anti-HER2), mesothelin (Biolegend, San Diego, CA, USA), and RP215 (Santa Cruz Biotechnologies, Dallas, TX, USA) were used as primary antibodies and labeled with FITC-conjugated polyclonal anti-human and anti-mouse IgG antibodies (Immunotools) for indirect staining.

### 2.5 Whole exome sequencing and microsatellite instability (MSI) score

The WES of the donor tumor tissue of HROP68 (primary and local recurrence as well as PDX models) was conducted by Centogene (Rostock, Germany) according to the protocol described by Trujillano and colleagues (17). In brief, DNA extraction was done using the QIAcube instrument with the QIAamp DNA Blood Mini QIAcube Kit (Qiagen, Hilden, Germany). In the exome, the target regions were amplified, and raw sequence data were analyzed. The HiSeq4000 platform (Illumina, San Diego, CA, USA) was used for sequencing. The used exome version was Centogene’s “CentoXome,” which covers approximately 33 Mb CCDS and is based on the Twist Bio Human Core Exome. Bioinformatic analysis was conducted as described by Matschos et al. (14). Pathogenetic classification was verified by an automated query to ClinVar (https://www.ncbi.nlm.nih.gov/clinvar/ accessed on 23.09.2022) followed by manual evaluation.

The MSI status of HROP68Tu2 was assessed using MSIsensor as described (18), a program that automatically detects somatic microsatellite changes, based on standard tumor-normal paired next- generation sequencing data. The number of somatic homopolymers and microsatellite sites is compared to the total number of sites. A percentage of 3.5%–20% somatic mutations is considered MSI-Low (MSI-L) and >20% MSI-High (MSI-H) (18).

### 2.6 *In vitro* drug response

The triplicates of HROP68Tu2 cells were seeded into a 96-well plate (1 × 10^4^). After 24 h, chemotherapeutics were added with a serial dilution technique, sparing healthy controls. The medium and drugs, respectively, were changed after 96 h to avoid deterioration effects. After 168 h, the medium was discarded, and cells were rinsed with PBS and analyzed by the crystal violet assay as described above. All assays were performed in independent triplicates. Lapatinib, PRIMA-1Met and olaparib (Hölzel Diagnostika, Köln, Germany), 5-FU, irinotecan, oxaliplatin, gemcitabine, paclitaxel, and erlotinib as single agents plus the combinations FOLFIRINOX, GemPac, and GemErlo in molar ratios analogous to the clinical regimen were tested (FOLFIRINOX: 1.07 folinic acid, 1.00 irinotecan, 80.95 5-FU, 0.80 oxaliplatin; GemPac: 1.00 gemcitabine, 0.04 paclitaxel (19). GemErlo: 1.00 gemcitabine, 0.21 erlotinib (20)). Individual interactions between 5-FU, irinotecan, and oxaliplatin were not assessed as monotherapy or other combinations are not recommended for clinical use. To evaluate the drug interactions, combination indices (CIs) (CI > 1.3 indicates antagonism, and CI = 0.6–0.8 indicates moderate synergism (19)) were calculated using the following equation (19):


$$\begin{array}{l} CI=\frac{IC50\;of\;combination\;\left(Drug\;1\;and\;2\right)\;relative\;to\;Drug\;1}{IC50\;of\;Drug\;1\;alone}\\ +\frac{IC50\;of\;combination\;\left(Drug\;1\;and\;2\right)\;relative\;to\;Drug\;2}{IC50\;of\;Drug\;2\;alone} \end{array}$$

### 2.7 Statistics

IC_50_ and IC_20_ values were calculated and analyzed by an unpaired Student’s t-test using Graph Pad Prism 5 (Graph Pad Software, San Diego, CA, USA). P-values ≤ 0.05 were considered statistically significant.

## 3 Results

### 3.1 Clinical case

**Figure 1:**
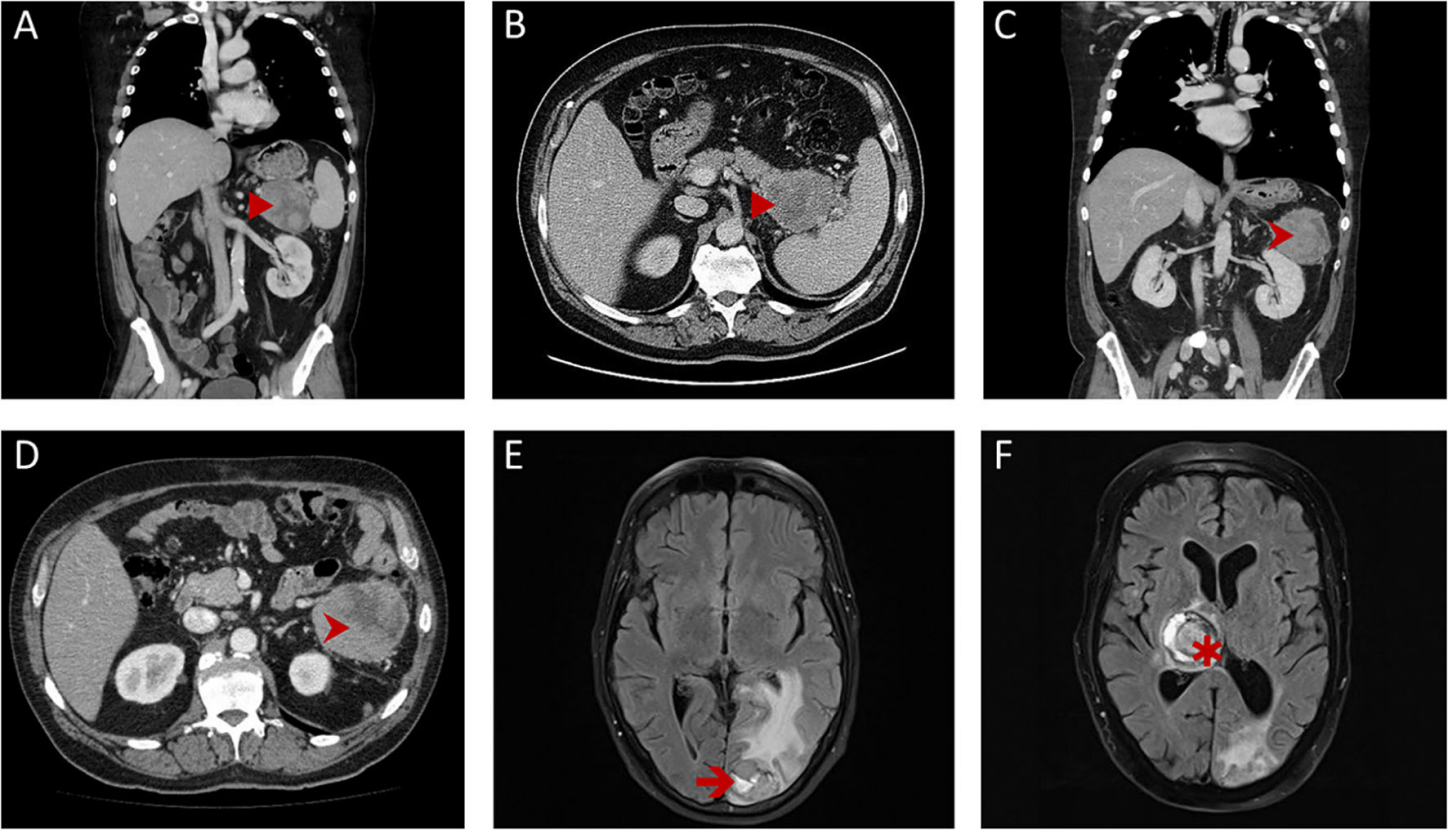
Radiological findings. First contrast-enhanced computed tomography (CEPT; **A**, **B**) of the thorax and abdomen revealed a large tumor (►) of the pancreatic tail, close to the splenic hilum with a pseudo-encapsulated appearance. Follow-up CEPT **(C, D)** showed isolated local recurrence (➤) with similar features in the left-upper abdomen and the retroperitoneum. Repeated magnetic resonance imaging scans of the brain showed a left occipital metastasis (➔) shortly after resection for local recurrence **(E)** and a rapidly progressing metastasis of the thalamus region (✱) over the course of palliative systemic treatment **(F)**.

A 64-year-old man presented with an excessive weight loss of 13 kg and fatigue during the last 5 months. Contrast-enhanced computed tomography (CEPT) revealed a large tumor of the pancreatic tail (**Figure 1**), and the patient was referred to our clinic for primary resection. He had no history of smoking, alcohol abuse, or prior cancer and neither did his first-degree relatives. Pancreatic left resection with splenectomy removed a soft and fragile, encapsulated tumor mass. Adjuvant treatment comprised of two cycles modified FOLFIRINOX, subsequently switched to five cycles of gemcitabine due to 5-FU-induced coronary vasospasm. Follow-up CEPT exhibited local recurrence in the left upper abdomen 11 months after resection. Since no distant metastasis was diagnosed, radical en bloc resection including the left colonic flexure and adrenal gland was performed. Pathological examination stated the completeness of resection and confirmed the origin of this second tumor from the previously resected MPC. The patient presented with severe disorientation and motor aphasia 2 weeks after discharge. Brain magnetic resonance imaging revealed occipital and parietal metastases, which were then treated by stereotactic radiotherapy. Subsequently, palliative systemic therapy was commenced, adding up to four cycles of gemcitabine plus nab-paclitaxel and one cycle of gemcitabine monotherapy in total. Although additional treatment with pembrolizumab was scheduled, the patient succumbed early to rapidly progressive cerebral metastases 23 months after the primary resection.

### 3.2 Pathology

**Figure 2:**
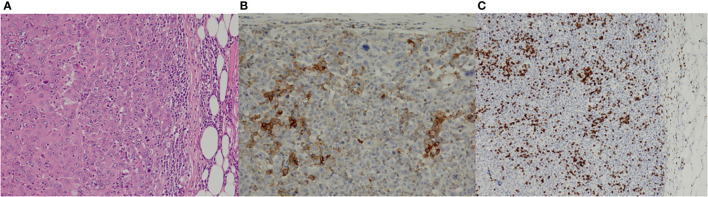
Pathological findings of the primary cancer. Hematoxylin–eosin stain (**A**; ×10) showed the solid sheets and nests of large polygonal neoplastic epithelial cells with a paucity of stromal components. Immunostaining for PD-L1 (**B**; ×20) and CD3 (**C**; ×10) reflected the immunogenic properties of the tumor.

The histologic examination of the primary tumor led to a diagnosis of MPC: the tumor was composed of solid sheets and nests of large polygonal neoplastic epithelial cells’ paucity of stromal components with polymorphous vesicular nuclei (**Figure 2**) in a stroma-poor background. Tumor borders were pushing and sharply delineated, and a brisk lymphocytic and histiocytic infiltrate was seen. A high mitotic index of 80%–90% and fairly large areas of tumor necrosis attested to a rapidly growing neoplasm. The tumor cells were immunohistochemically positive using a pan-cytokeratin cocktail (AE1/3) as well as Ber-EP4 and CK7 positive, whereas CK20, CDX2, HMB45, Hepar-1, LCA, and AFP immunostains were negative. Mismatch repair protein MLH1 and MSH2 expression was retained, molecular MSI testing (Bethesda markers plus the mononucleotide marker CAT25 (21) additionally confirmed a microsatellite-stable tumor. PD-L1 (clone 22C3) immunostaining was positive in approximately 20% of the tumor cells, albeit membranous immunostaining was weak, mostly; the tumor-infiltrating lymphocytes and histiocytes were PD-L1 positive throughout. This allowed the conclusion that this MPC was immunogenic.

### 3.3 Patient-derived xenograft establishment

**Figure 3:**
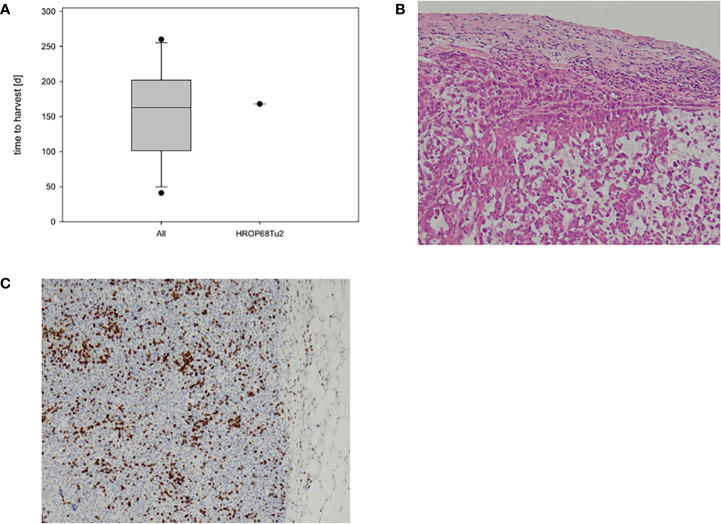
HROP68Tu2 PDX. Growth kinetics of HROP68Tu2 **(A)** compared to other pancreatic PDXs with successful outgrowth in passage fT0 (n = 12). The *in vivo* growth of HROP68Tu2 did not differ from the median growth rate of other pancreatic PDX. The hematoxylin–eosin stain of the PDX (B; 20x) revealed a close resemblance to the original donor tumor **(B)**. Tumor cells showed strong immunostaining for PD-L1 **(C)**.

Successful engraftment, defined as tumor outgrowth to a target volume of 1,500 mm^3^, was observed after the implantation of the primary resected tumor (HROP68Tu1) as well as the local recurrence (HROP68Tu2). In both cases, two out of two mice showed tumor outgrowth in at least one flank. HROP68Tu2 reached the target size 168 days after implantation. The growth kinetics of HROP86Tu2 compared well to those of other successfully engrafted pancreatic PDXs (n = 12) established from our group (**Figure 3A**). To confirm histopathological congruency with the original tumor and to exclude a potential murine lymphoma, a common pitfall in xenograft development, histologic sections (hematoxylin–eosin and PD-L1 immunostaining) were reviewed by an experienced pathologist (**Figures 3B, C**).

### 3.4 Morphology, growth kinetics, and migration of HROP68Tu2 cell line

**Figure 4:**
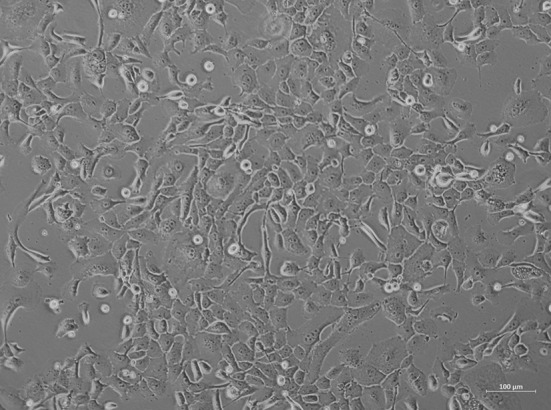
Cell morphology of the primary cell line HROP68Tu2 in passage 11. Picture was taken using an inverted microscope at ×40 magnification.

A permanent 2-dimensional (2-D) cell line was established by immediate incubation of freshly resected tissue obtained from the secondary surgery (HROP68Tu2) as described before (22). After adaption to standard culture conditions, HROP68Tu2 cells were passaged more than 40 times as a permanent cell line. Cells were regularly tested negative for mycoplasma contamination. Cross-contamination or mix-up was excluded by STR analysis, confirming the donor–patient origin. HROP68Tu2 cells grew evenly as an adherent monolayer with heterogeneous cell size and morphology (**Figure 4**). Doubling time in the exponential growth phase was 89.56 h ( ± 17.46). Morphology and growth did not change significantly during 40 consecutive passages.

**Figure 5:**
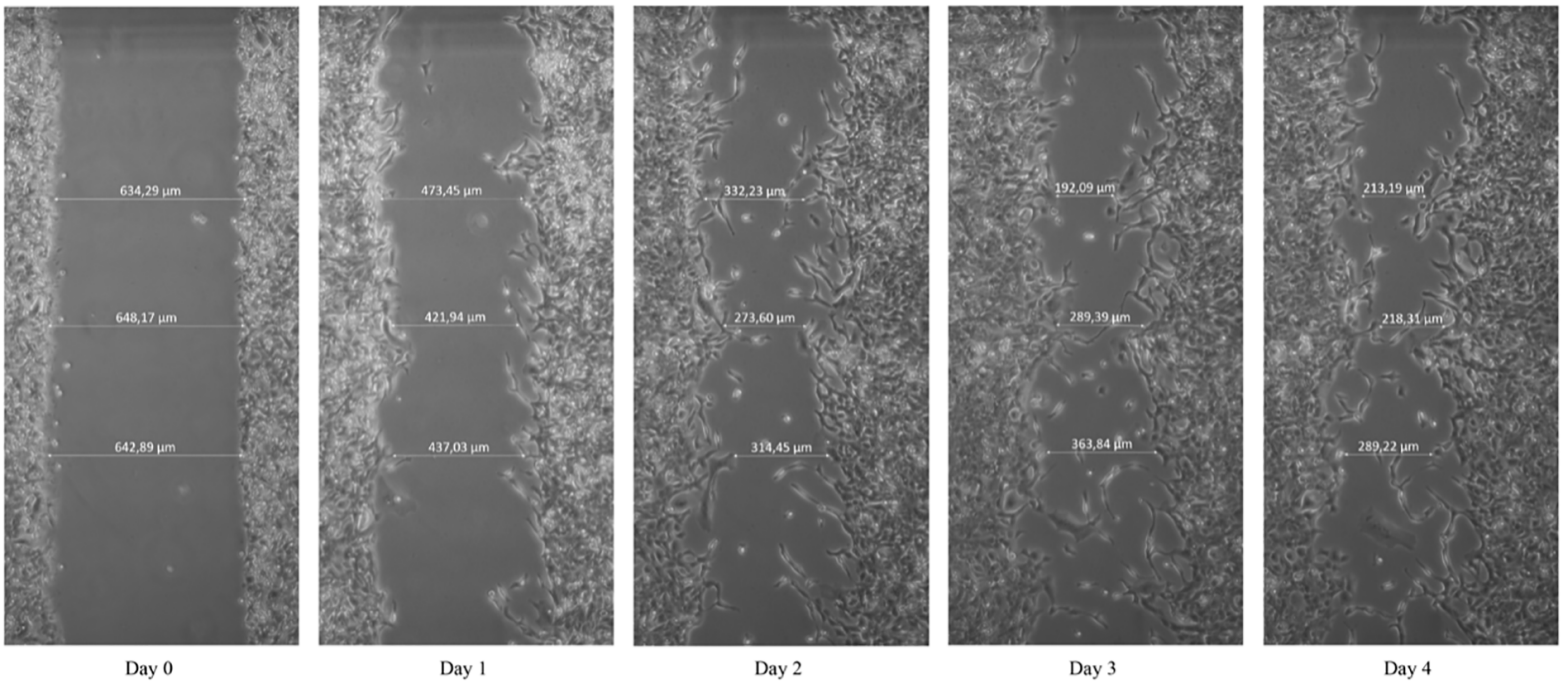
Scratch assay. Cells were scratched using a 10 µl pipet tip. Pictures were taken using an inverted microscope at ×10 magnification.

Cells migrated with 1.78 µm/h ( ± 0.84) in a classical scratch assay. The migratory behavior appeared to be invasive as cells detached from the scratch’s edges and migrated into the scratch as single cells (**Figure 5**).

### 3.5 Flow cytometric characterization

**Figure 6:**
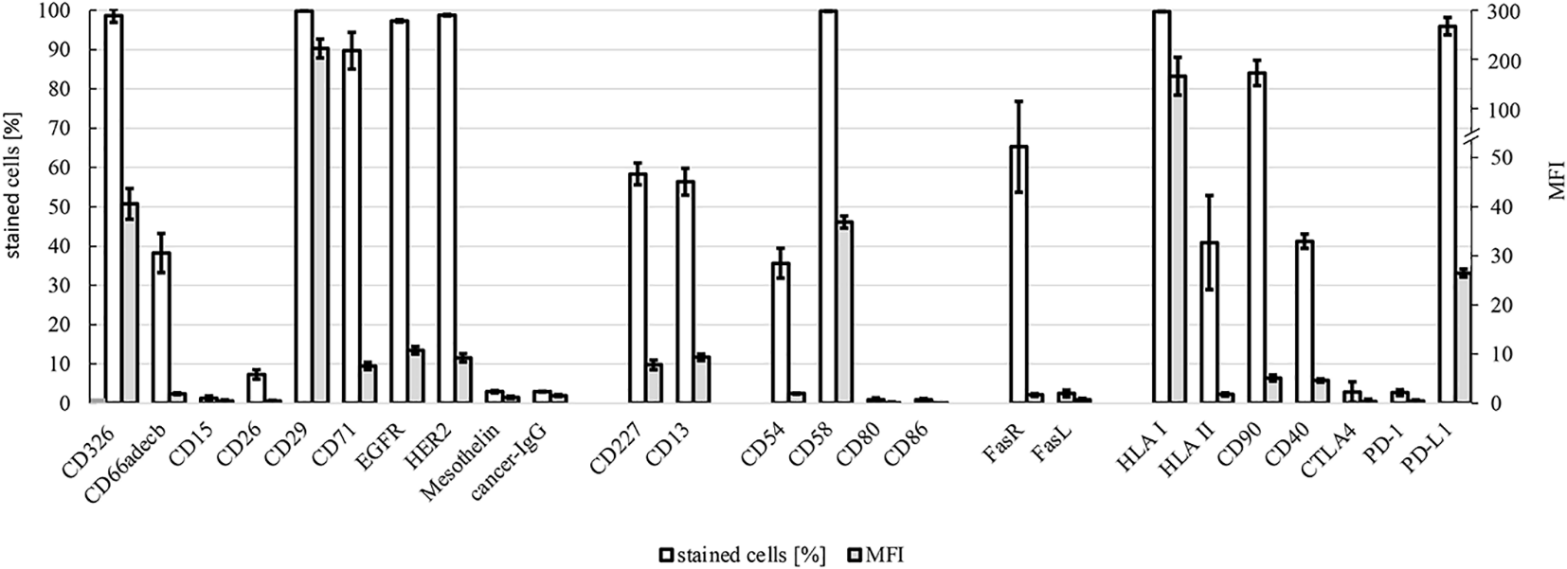
Flow cytometry. Expression of surface markers in HROP68Tu2 cells: tumor markers/signaling/proliferation (CD326, CD66adecb, CD15, CD26, CD29, CD71, EGFR, HER2, mesothelin, and cancer IgG), drug resistance (CD227 and CD13), adhesion/cell–cell interaction (CD54, CD58, CD80, and CD86), death ligand and receptor (CD95 and CD178), and immunogenicity/immunosuppression (HLA I, HLA II, CD90, CD40, CTLA4, PD-1, and PD-L1) were assessed by flow cytometry using FACS Calibur. Positively stained cells are given in % ± SEM and MFI (mean fluorescence intensity) ± SEM.

HROP68Tu2 cells´ expressions of surface proteins were evaluated by flow cytometry (**Figure 6**). Common epithelial and tumor markers were expressed to a variable extent: CD326 (EpCAM) (98.6% ± 1.7, MFI: 40.6 ± 3.1), CD227 (MUC-1) (58.4% ± 2.8, MFI: 7.8 ± 0.1), and CD66adecb (CEA) (38.2% ± 0.2, MFI: 5.0 ± 0.2). However, the epithelial markers CD15 (1.3% ± 0.6, MFI: 0.4 ± 0.3) and CD26 (7.3% ± 1.2, MFI: 0.4 ± 0.2) could not be detected. The adhesion marker CD29 (Integrin *β* - 1) was highly expressed (99.81% ± 0.01, MFI: 221.28 ± 19.23). A common pattern of the Fas receptor (CD95) (65.3% ± 11.6, MFI: 1.7 ± 0.3) and aberrant Fas ligand (CD178) (2.5% ± 0.9, MFI: 0.7 ± 0.3) expression was detected. HLA I expression was preserved homogeneously and with high intensity (99.7% ± 0.1, MFI: 165.6 ± 38.4). An average of 40.9% (± 12.0) of HROP68Tu2 cells expressed HLA II, albeit with low intensity (MFI: 1.8 ± 0.3). Furthermore, the cells expressed several proteins that are targets of antibody-based therapies. Matching the pathology report of the patient tumor, but even more so of the PDX, HROP68Tu2 cells showed homogeneous and high PD-L1 expression (95.9% ± 2.2, MFI: 26.5 ± 0.8). Furthermore, CD90 (84.0% ± 3.2, MFI: 5.1 ± 0.6) and CD40 (TNFRS5) (41.3 ± 1.83, MFI: 4.6 ± 0.3) expression was observed.

### 3.6 Whole exome sequencing: Somatic mutations and MSI scoring

The overall number of somatic mutations was determined by the WES analysis of HROP68 tumor tissues (primary cancer and local recurrence), subtracting alterations from the reference genome observed in the normal DNA of the patient. In sum, there was a relatively high TMB with 486 somatic mutations for HROP68Tu1 and 729 for HROPTu2, as well as an even higher number of 1,498 for HROP68Tu1 T1 M2 and 1,964 for HROP68Tu2 T0 M2. The higher numbers on the PDX models likely reflect the high tumor cell purity and possibly the very short ischemic time between collection and snap freezing. No mutations were observed in the following MMR genes: *MLH1, MSH2, MSH6, PMS2*, and *EPCAM (CD326)*. In addition, we discovered a non-coding *POLD1* mutation in the PDX tissue of HROP68Tu2. Furthermore, the WES revealed a high amount of frameshift mutations, a total number of 92 in the primary tumor and 113 in the local recurrence, HROP68Tu1 T1 M2: 192 and HROP68Tu2 T0 M2: 154.

As can be depicted from **Table 1**, several mutations classified as pathogenic affecting tumor-relevant genes including *KRAS* and *TP53* could be identified. The mutations in *BRCA1* and *TP53* were subsequently included as targets in a precision oncology fashion sensitivity testing.

**Table 1:** Mutational profiling of the HROP68 primary tumor (HROP68Tu1), HROP68 recurrent tumor (HROP68Tu2), and their PDX models (HROP68Tu1 T1 M2 and HROP68Tu2 T0 M2).

Gene	Ref	Alt	Type	Coding Pep	dbSNP ID	HROP68Tu1	HROP68Tu1 T1 M2	HROP68Tu2	HROP68Tu2 T0 M2
*BRCA1*	T	C	missense	Asn277Asp	rs2054040121				15
*CDKN2A*	G	A	missense	Pro75=	rs762397298				38
*FOXG1*	CG	C	in/del	Glu154fs	rs398124204	54	73	49	45
*GRIK2*	C	T	stop	Arg198*	rs749995448		17		
*KAT6B*	T	C	missense	Ile413Thr	rs1842099343		25		31
*KRAS*	C	T	missense	Gly12Asp	rs121913529	18	30	35	24
*RUNX1*	A	C	missense	Ser424Ala	rs2056451534		24		20
*SMAD4*	T	A	synonymous	Pro303	rs141149381		17		
*TP53*	C	A	missense	Cys277Phe	rs763098116	95	98	87	100

Given are somatic mutations affecting tumor-relevant genes as determined by WES. Also displayed are reference (Ref) and alternative (Alt) nucleobases, the type of mutation, effects on the protein sequence (Coding Pep), dbSNP IDs, and the variant allele frequencies in %. * marks a premature stop codon.

Moreover, WES data were also used to calculate an MSI score, which was determined by MSIsensor to be 16.28% for the primary tumor and 13.77% for HROP68Tu2. In comparison: 23 other pancreatic cancer cases in our biobank had a mean MSI score of 3.09 (range 0.52 to 6.38). Taking the limit of MSIsensor for MSI-H of at least 20% into account, this analysis confirmed the absence of MSI-H, thereby matching the pathological findings. Still, when considering the relatively high TMB, the overall number of mutations reflecting frameshifts in coding genomic regions as identified by MSIsensor analysis, we consider HROP68 as an MPC with a low level of MSI (MSI-L).

The WES data obtained from Centogene as. vcf files are available upon reasonable request.

### 3.7 *In vitro* drug response

The treatment response of HROP68Tu2 was tested toward selected clinically approved therapeutics and several novel targeted drugs selected according to the observed somatic mutations and membrane receptors expressed by HROP68Tu2 cells. These were lapatinib due to the EGFR and HER2 expression, PRIMA-1Met because of the TP53 mutation, and, similarly, olaparib due to the BRCA1 mutation. HROP68Tu2 cells were sensitive toward all tested drugs within the therapeutic range as defined by achievable human plasma levels (**Table 2**).

**Table 2:** Chemosensitivity of HROP68Tu2 toward single drugs: IC_50_ and IC_20_ values.

	5-FU	Irinotecan	Oxaliplatin	Gemcitabine	Paclitaxel	Erlotinib	Lapatinib	PRIMA-1Met	Olaparib
IC_50_	6.83 (± 2.52)	0.32 (± 0.06)	1.55 (± 0.74)	0.00057 (± 0.00016)	0.0070 (± 0.00162)	6.52 (± 1.72)	6.27 (± 2.96)	23.98 (± 5.40)	6.69 (± 1.9)
IC_20_	1.18 (± 33.33)	0.09 (± 0.01)	0.30 (± 0.15)	0.00024 (± 0.00024)	0.0024 (± 0.00142)	2.99 (± 0.86)	1.87 (± 2.36)	17.94 (± 4.88)	1.35 (± 0.09)
C_max_	384.40 (23)	17.04 (24)	5.03 (25)	98.3 (26)	5.10 (27)	10.20 (28)	14 (29)	250,000 (30)	13.1 (26)

IC_50_ and IC_20_ values of HROP68Tu2 are given in µM for the single therapeutic drugs tested. To allow the integration of the data into a clinical context, the maximum plasma concentrations (C_max_ [µM]), reported in patients undergoing chemotherapy, were supplemented (references in brackets).

Next, the response to the clinically used combination therapy FOLFIRINOX was analyzed. Although a moderate synergistic effect of the drugs 5-FU, irinotecan, and oxaliplatin was observed, chemosensitivity to FOLFIRINOX compared to 5-FU alone was not significantly increased (p = 0.118). The interaction of the drugs involved can be judged by calculating a combination index (CI), which was in this case 0.71, thereby indicating a moderate synergistic effect for FOLFIRINOX (**Table 3**).

**Table 3:** Drug interaction of FOLFIRINOX.

FOLFIRINOX							
IC50 values in µM						Interaction	
5-FU	FOLFIRINOX (Relative to 5-FU)	Irinotecan	FOLFIRINOX (Relative to Irinotecan)	Oxaliplatin	FOLFIRINOX (Relative to oxaliplatin)	CI value	
6.83	3.70	0.32	0.05	1.55	0.04	0.71	moderate synergism

IC_50_ values of HROP68Tu2 are given for the single drugs and for the FOLFIRINOX combinations, respectively, in µM. The IC_50_ values of FOLFIRINOX are given as relative to the corresponding single drug, based on the molar ratios (2.6). The combination index (CI) and the resulting conclusion of moderate synergism between the components of the regimen are also given.

Contrary, combining gemcitabine with paclitaxel or erlotinib did not improve inhibitory effects compared to gemcitabine alone but rather showed antagonistic effects between gemcitabine and paclitaxel (GemPac; CI = 1.44, p>0.05) as well as gemcitabine and erlotinib (GemErlo; CI = 1.53, p>0.05) (**Table 4**).

**Table 4:** Drug interaction of GemPac and GemErlo.

GemPac						
IC50 values in nM					Interaction	
Gemcitabine	GemPac (Relative to Gemcitabine)	Paclitaxel	GemPac (Relative to Paclitaxel)		CI value	
0.57	0.82	7.04	0.01		1.44	Antagonism
GemErlo						
IC50 values in nM					Interaction	
Gemcitabine	GemErlo (Relative to Gemcitabine)	Erlotinib	GemErlo (Relative to Erlotinib)		CI value	
0.57	0.87	652	0.18		1.53	Antagonism

IC50 values are given for the single drugs and for the GemPac and GemErlo combinations, respectively in nM. The IC_50_ values of GemPac and GemErlo are given as relative to the corresponding single drug, based on the molar ratios (2.6). The combination index (CI) and the resulting conclusion of antagonism between the components of the regimen are also given.

## 4 Discussion

Patient-derived tumor models are the cornerstone of preclinical and translational research (14, 15, 22) and are valuable tools for the evaluation of novel drugs or treatment options for rare diseases (16, 31). The consequent biobanking of all resected pancreatic cancers at our department provided the opportunity to seize this rare case of primary and recurrent MPC, as well as to establish PDX from both the surgical specimen and a PDCL. The establishment of stable cell lines derived directly from the fresh surgical PDAC specimen is rarely successful, which can be mostly attributed to a low tumor–stroma ratio frequently causing fibroblastic overgrowth (32, 33). The positive model establishments of HROP68 might thus be mostly attributable to the high tumor–stroma ratio diagnosed in this case.

Generally, the evaluation of treatment efficacy for the individual PDAC patient is challenging since long-term survival remains an exception. This is explained by the intrinsic chemoresistance of PDAC, attributable to the large and dense tumor stroma, decreasing microvascularity and thereby drug delivery, to the epithelial cancer cell nests embedded into this stroma (34, 35). Due to the paucity of stromal components in HROP68, this relationship is unlikely in this case and might explain the observed drug responses of the present study. Compared to published data from many long-term established PDAC cell lines, HROP68Tu2 cells were highly responsive to gemcitabine but only moderately to 5-FU. Moreover, a moderate synergism was observed between the components of the FOLFIRINOX regimen. Contrary to that, an antagonistic interaction was observed for the combination of gemcitabine with both paclitaxel and erlotinib. However, due to the rareness of MPC, the comparison of these results with clinical data or even the retrospective translation of the presented case into the clinical context is hardly possible.

PDAC are bona fide non-immunogenic due to their low TMB; only 1%–2% of PDAC exhibit high levels of MSI, which is typically associated with a hypermutated phenotype (36). However, the associations of MSI to a medullary and mucinous histology have been described (37). Despite the high level of T-cell infiltration, the hallmark of ongoing immune recognition, observed in HROP68, the case was diagnosed as MSS both by immunostaining with a retained expression of the mismatch repair proteins MLH1 and MSH2, as well as by molecular microsatellite stability testing. Somehow challenging this diagnosis, WES analysis, however, identified a proportionally large number of somatic mutations, including a relevant fraction of insertions and deletions leading to frameshift mutations in coding regions. The latter translates to an elevated score in the MSIsensor analysis, albeit not reaching the 20% threshold to allow formally judging the case as MSI-H. Mutations of other candidate genes associated with a high TMB, like *POLE* (38), *POLD1* (39), and *MUTYH* (40), could not be detected in the clinical samples, although the PDX HROP68Tu2 T0 M2 harbored a *POLD1* mutation of uncertain significance. By applying a less rigorous tertiary data interpretation of the WES analysis, likely pathogenic mutations of DNA repair genes, known to be associated with an increased TMB and immunogenicity, like PALB2, ATR, and CHECK2 (41–43), were identified. However, when summing up all pieces of information, the uncommonly high TMB (44), combined with the MSI score, led us to the conclusion that HROP68 is an MSI-L MPC.

Although mutations of *BRCA1/2* in PDAC are associated with increased survival due to a higher susceptibility to platinum-based chemotherapy (45, 46), this observation could not be confirmed for HROP68, harboring a *BRCA1* mutation. While *BRCA*-mutated ovarian cancers respond well to the inhibitors of the poly-ADP ribose (PARPi), the PARPi olaparib improved progression-free survival in metastatic PDAC patients with a germline *BRCA* mutation but failed to improve overall survival (47, 48). In our study, olaparib had an inhibitory effect on HROP68Tu2 cells *in vitro*.

Similarly successful, HROP68Tu2 cells showed marked susceptibility to the tyrosine kinase inhibitors erlotinib and lapatinib, which were selected as targeted agents due to the homogeneous and high expression of EGFR and HER2. In the clinical setting, erlotinib led to small survival benefits, but lapatinib recently failed to improve the survival of patients with metastatic PDAC (49, 50).

The *TP53* mutation displayed by HROP68 is classified as uncertain significance in the ClinVar database. However, it might be pivotal regarding the proliferation of HROP68 cells since it replaces a cysteine on the protein level and this, together with the loss of the second allele, likely results in a loss of proper protein function. Our data of the PRIMA-Met1 treatment response support this hypothesis since this drugs’ ability to restore wild-type p53 conformation has been shown for several mutant p53 proteins (51). The clear impact of PRIMA-Met1 on HROP68Tu2 cells would thus deliver evidence for the pathogenic character of the Cys277Phe p53 mutation with the dbSNP-ID rs763098116.

The immune landscape and interplay between various cell types contributing to immune evasion and a pro-inflammatory microenvironment are complex and not fully understood (52). On one hand, HROP68Tu2 showed a robust and homogeneous expression of HLA I as well as the expression of HLA II, CD58 (LFA3), and CD54 (ICAM1), allowing recognition by antigen-specific immune cells. On the other hand, the homogenous expression of PD-L1, CD90, and CD40 probably facilitated T-cell inactivation and likely immune evasion. PD-L1 expression is uncommon in PDACs and can only be found in 3% of cases (53). Although Le et al. (54) reported promising results for the efficiency of the PD-L1-inhibitor pembrolizumab for the treatment of different solid, MSI-positive tumors, the tumor response to pembrolizumab in MSI-positive PDCAs was rather modest with 18% in the KEYNOTE-158 Study (54, 55). Due to the uncommon combination of proteins expressed by HROP68 tumor cells, we consider it likely that an early application of checkpoint inhibitors in the clinical course of this patient might have improved the outcome, at least the overall survival time. It is one of the weak points of the current study that autologous immune analyses have not (yet) been performed *in vitro* using the HROP68Tu2 cell line and the peripheral as well as tumor-infiltrating T cells of the patient. Such analyses are planned, but, due to the restricted number of T cells available in our biobank, they shall be combined with an analysis of tumor-specific antigenic epitopes presented by HROP68Tu2 cells.

In summary and to the best of our knowledge, the PDX and PDCL of HROP68Tu2 are the first preclinical models established from an MPC. Although their preclinical implications are limited by the rarity of MPC, they are perfect tools for the establishment of novel drugs in the context of this rare subset of PDAC. Moreover, the observed good *in vitro* responses toward several drugs tested also emphasize the necessity to evaluate therapeutic options on a patient–individual level, i.e., precision oncology, in order to optimize the clinical outcome, especially for rare cancer cases.

## Data availability statement

The original contributions presented in the study are publicly available. The data has been uploaded to the NCBI BioProject repository and can be found using accession number PRJNA915478.

## Ethics statement

The studies involving human participants were reviewed and approved by Institutional review board of the University Medical Center Rostock (A 2018-0054). The patients/participants provided their written informed consent to participate in this study. The animal study was reviewed and approved by Landesamt für Landwirtschaft, Lebensmittelsicherheit und Fischerei Mecklenburg-Vorpommern.

## Author contributions

Design and conception of the study: ML. Patients’ consent and clinical material acquisition: FB, ML, CM, and FP. Experiments: JvdD, JF, FB, and FP. Manuscript draft: JvdD, JF, FB, FP, CM, and ML. All authors contributed to the article and approved the submitted version.
